# Unveiling complexity: non-linear and fractal analysis in neuroscience and cognitive psychology

**DOI:** 10.3389/fncom.2014.00017

**Published:** 2014-02-21

**Authors:** Tobias A. Mattei

**Affiliations:** Department of Neurological Surgery, The Ohio State University Medical CenterColumbus, OH, USA

**Keywords:** non-linear analsyis, complex systems, fractal analysis, cognitive psychology, neurosciences

Although non-linear dynamics has been mastered by physicists and mathematicians for a long time, as most physical systems are inherently non-linear in nature (Kirillov and Dmitry, 2013), the more recent successful application of non-linear and fractal methods to modeling and prediction of several evolutionary, ecologic, genetic, and biochemical processes (Avilés, 1999) has generated great interest and enthusiasm for such type of approach among researchers in neuroscience and cognitive psychology.

After initial works on this emerging field, it became clear that that multiple aspects of brain function as viewed from different perspectives and scales present a nonlinear behavior, with a complex phase space composed of multiple equilibrium points, limit cycles, stability regions, and trajectory flows as well as a dynamics which includes unstable periodic orbits, period-doubling bifurcations, as well as other features typical of chaotic systems (Birbaumer et al., 1995). Moreover it was also demonstrated that non-linear dynamics was able to explain several unique features of the brain such as plasticity and learning (Freeman, 1994).

More recently the concept of strange attractors has lead to a new understanding of information processing in the brain which, instead of the old “localizationist” approaches (Wernicke, 1970), considers higher cognitive functions (such as language, attention, memory and decision-making) as systemic properties which emerge from the dynamic interaction between parallel streams of information flowing between highly interconnected neuronal clusters that are organized in a widely distributed circuit modulated by key central nodes (Mattei, 2013a,b). According to such paradigm, the concept of self-organization has been able to offer a proper account of the phenomenon of evolutionary emergence of new complex cognitive structures from non-deterministic random patterns, similarly to what has been previously observed in nonlinear studies of fluid dynamics (Dixon et al., 2012).

Additionally, the challenges of interpreting massive amounts of information related to brain function generated from emerging research fields in experimental neuroscience (such as functional MRi, magnetoencephalography, optogenetics, and single-cell intra-operative recordings) have generated the necessity of new methods for which incorporate complex pattern analysis as an important feature of their algorithms (Turk-Browne, 2013).

Up to now nonlinear methods have already been successfully employed to describe and model (among many other examples) single-cell firing patterns (Thomas et al., 2013), neural networks synchronization (Yu et al., 2011), autonomic activity (Tseng et al., 2013), electroencephalographic data (Abásolo et al., 2007), noise modulation in the cerebellum (Tokuda et al., 2010), as well as higher cognitive functions and complex psychiatric disorders (Bystritsky et al., 2012). Additionally fractal analysis has been extensively explored not only in the description of the temporal aspects of neuronal dynamics, but also in the evaluation of key structural patterns of cellular organization in both normal and pathological histologic brain samples (Mattei, 2013a,b).

Finally, recent studies have demonstrated that several cognitive functions can be successfully modeled with basis on the transient activity of large-scale brain networks in the presence of noise (Rabinovich et al., 2008). In fact, it has already been suggested that the observed pervasiveness of the 1/f scaling (also called 1/*f* noise, fractal time, or pink noise) in both neural and cognitive functions may have a very close relationship (if not a causal one) with the phenomenon of metastability of brain states (Kello et al., 2008). Other studies in the emerging field of neuroeconomics have shown that it is possible to represent typical decision-making paradigms by dynamic models governed by ordinary differential equations with a finite number of possibilities at the decision points as well as basic rules to address uncertainty (Holmes et al., 2004).

In this special edition of Frontiers Computational Neuroscience dedicated to the topic of Non-linear and Fractal Analysis in Neuroscience and Cognitive Psychology, special articles from several frontline research groups around the world were carefully selected in order to provide a representative sample of the different research fields in neuroscience and cognitive psychology where non-linear and fractal analysis may be successfully applied.

The selected articles include both classical problems where non-linear method have been traditionally employed (such as EEG data analysis) as well as other new research fields in which non-linear analysis has been shown to be useful not only for modeling normal brain dynamics but also for the diagnosis of neurological and psychiatric disorders, monitoring of their natural history and evaluation of the effects of different therapeutic strategies.

Overall, both theoretical and experimental works in the field seem to demonstrate that the advanced tools of non-linear analysis can much more accurately describe and represent the complexity of brain dynamics than traditional mathematical and computational methods based on linear and deterministic analysis.

Although it seems quite unquestionable that future attempts to model complex brain and cognitive functions will significantly benefit from non-linear methods, the exact cognitive and neuronal variables that may exhibit a significant chaotic pattern is still an open question. However, taking into account the pervasiveness of non-linear behavior in the brain, which has already been demonstrated by such an extensive literature in so many different fields of neuroscience and cognitive psychology (as well as the remarkable progress that has been achieved by the application of non-linear and fractal analysis in such research areas), maybe the burden of proof should be on the other side. Perhaps the real question to be answered is: Which areas of neuroscience and cognitive psychology would not benefit from the advantages that non-linear and fractal analysis has to offer?
